# RETRACTION: A Novel Model of Cancer‐Induced Peripheral Neuropathy and the Role of TRPA1 in Pain Transduction

**DOI:** 10.1155/prm/9768981

**Published:** 2026-11-06

**Authors:** Pain Research and Management

RETRACTION: A. Maqboul and B. Elsadek, “A Novel Model of Cancer‐Induced Peripheral Neuropathy and the Role of TRPA1 in Pain Transduction,” *Pain Research and Management* (2017): 3517207, https://doi.org/10.1155/2017/3517207.

The above article, published online on 28 December 2017 in Wiley Online Library (https://onlinelibrary.wiley.com), has been retracted by agreement between the journal Editor‐in‐Chief, Massimiliano Valeriani, and John Wiley & Sons Ltd. An Expression of Concern was previously published for this article on 10 December 2018 (https://doi.org/10.1155/2018/5342101) which detailed that this article was under investigation by the Faculty Board of the Charité – University Medical Center Berlin, and in December 2020 it was updated to note that the research reported therein had been subject to legal proceedings at the Verwaltungsgericht Berlin (Berlin Administrative Court).

The Publisher has been informed by representatives of the Charité – University Medical Center Berlin that their investigation has concluded. Additionally, the legal proceedings at the Berlin Administrative Court have concluded. The investigation found that there were significant concerns over the published data, the reported methods, and the materials described in the paper, as well as missing authorship. Therefore, all parties agree that the article must be retracted. Author A. Maqboul disputed the decision to issue the retraction. Author B. Elsadek did not correspond regarding the retraction.

